# The Mitochondrial Proteomic Signatures of Human Skeletal Muscle Linked to Insulin Resistance

**DOI:** 10.3390/ijms21155374

**Published:** 2020-07-28

**Authors:** Rikke Kruse, Navid Sahebekhtiari, Kurt Højlund

**Affiliations:** 1 Steno Diabetes Center Odense, Odense University Hospital, DK-5000 Odense C, Denmark; rkruse@health.sdu.dk (R.K.); nsahebekhtiari@health.sdu.dk (N.S.); 2 Department of Clinical Research & Department of Molecular Medicine, University of Southern Denmark, DK-5000 Odense C, Denmark

**Keywords:** mitochondrial proteomics, mitochondria, skeletal muscle, insulin resistance, Type 2 diabetes

## Abstract

Introduction: Mitochondria are essential in energy metabolism and cellular survival, and there is growing evidence that insulin resistance in chronic metabolic disorders, such as obesity, type 2 diabetes (T2D), and aging, is linked to mitochondrial dysfunction in skeletal muscle. Protein profiling by proteomics is a powerful tool to investigate mechanisms underlying complex disorders. However, despite significant advances in proteomics within the past two decades, the technologies have not yet been fully exploited in the field of skeletal muscle proteome. Area covered: Here, we review the currently available studies characterizing the mitochondrial proteome in human skeletal muscle in insulin-resistant conditions, such as obesity, T2D, and aging, as well as exercise-mediated changes in the mitochondrial proteome. Furthermore, we outline technical challenges and limitations and methodological aspects that should be considered when planning future large-scale proteomics studies of mitochondria from human skeletal muscle. Authors’ view: At present, most proteomic studies of skeletal muscle or isolated muscle mitochondria have demonstrated a reduced abundance of proteins in several mitochondrial biological processes in obesity, T2D, and aging, whereas the beneficial effects of exercise involve an increased content of muscle proteins involved in mitochondrial metabolism. Powerful mass-spectrometry-based proteomics now provides unprecedented opportunities to perform in-depth proteomics of muscle mitochondria, which in the near future is expected to increase our understanding of the complex molecular mechanisms underlying the link between mitochondrial dysfunction and insulin resistance in chronic metabolic disorders.

## 1. Introduction

Mitochondria are ubiquitous cellular organelles that, except in erythrocytes, determine the metabolic activity of tissues and capture the chemical energy of food to form ATP through coupling of cellular respiration to oxidative phosphorylation (OXPHOS). Mitochondria are essential in energy metabolism and for cellular survival and play a central role in numerous metabolic processes, such as OXPHOS, the tricarboxylic acid (TCA) cycle, β-oxidation, amino acid metabolism, apoptosis, heme synthesis, steroid synthesis, and calcium homeostasis. In addition to energy, OXPHOS also generates reactive oxygen species (ROS) [1,2,3]. It is therefore not surprising that impaired mitochondrial function is associated with a vast array of human disorders characterized by metabolic perturbations, including obesity, type 2 diabetes (T2D), cancer, cardiovascular disease, myopathies, and neurodegenerative diseases [1,2,3,4,5,6,7].

Insulin resistance (IR) plays a key role in the development of T2D, which is one of the greatest global health challenges, with an estimated 463 million people having diabetes in 2019 [8]. Despite intense research, the precise molecular mechanisms underlying the pathogenesis of IR are still not fully understood. Nonetheless, as key players in energy metabolism, the content and function of mitochondria have been the subject of numerous studies in obesity and T2D-related research [9,10,11,12,13,14,15] and multiple alterations in mitochondrial content, structure, and function have been reported in skeletal muscle, heart, liver, and adipose tissue in insulin-resistant conditions, such as polycystic ovary syndrome [16], obesity, T2D, and aging [9,10,11,12,13,14,15,17,18,19,20]. In particular, several abnormalities in mitochondrial oxidative metabolism, including reduced content and size of mitochondria [11], impaired mitochondrial respiration, and reduced electron transport chain activity [11,12,17], as well as transcriptional downregulation of genes involved in mitochondrial oxidative metabolism [13,14,19], have been associated with obesity, T2D, and aging in human skeletal muscle. These alterations are collectively termed mitochondrial dysfunction and are often associated with an increased intramyocellular lipid content [9] and with an impaired insulin-mediated increase in transcript levels, protein abundance, and ATP synthesis in the mitochondria of skeletal muscle in insulin-resistant individuals [9,21].

Skeletal muscle plays a prominent role in the metabolic homeostasis as it is responsible for all voluntary body movements and is a primary target and modulator of the beneficial effects of exercise [22]. In both healthy lean and obese individuals and in patients with T2D, exercise training improves insulin sensitivity [20,23,24,25,26] and increases the mitochondrial oxidative capacity in skeletal muscle by enhancing mitochondrial biogenesis [20,27,28], mainly through activation of peroxisome proliferator-activated receptor-γ co-activator (PGC)-1α [29,30,31]. Skeletal muscle, which accounts for 40% of the total body weight, is the major site of insulin-stimulated glucose uptake [32,33] and as such, skeletal muscle is quantitatively the predominant site of peripheral IR [34]. Collectively, there is clear evidence of an association between skeletal muscle IR and abnormalities in mitochondrial oxidative metabolism, although it remains to be established whether mitochondrial dysfunction is a cause or consequence of IR, or both [35,36].

Mass spectrometry (MS) is a potent tool in the identification and characterization of the proteomes of organs and subcellular compartments, including mitochondria in both healthy and pathophysiological conditions. Therefore, it is not surprising that a large portion of the life science research over the past decades has taken advantage of this ever-developing technique to increase the understanding of proteins and their functions. The technical and methodological advances in the field of proteomics now allow a deeper characterization of the proteome as well as of post-translational modifications (PTMs), thus enabling a more comprehensive characterization of the mitochondrial proteome in human skeletal muscle. Simultaneous quantitative analysis of thousands of proteins, which is ultimately the aim of many proteomic studies, provides the molecular signature of any given disease. Considering the pivotal role of mitochondria in cell metabolism and the presence of mitochondrial dysfunction in skeletal muscle of aging and disorders, such as obesity and T2D, the identification of novel mechanisms that control mitochondrial metabolism in skeletal muscle may provide new concepts to enhance mitochondrial oxidative metabolism and prevent and/or treat these disorders.

In the present review, we aim to address the current strategies for characterization of the mitochondrial proteome and to summarize the existing knowledge of the proteomic signatures of human skeletal muscle mitochondria in relation to insulin-resistant conditions, such as obesity, T2D, and aging, as well as the effects of exercise, a well-known counteractor of IR. To narrow the scope of the review, we will only refer to studies of human skeletal muscle unless otherwise stated.

## 2. Technical Considerations in Skeletal Muscle Proteomics

The field of metabolic research is currently expanding dramatically because many different diseases are being characterized by changes in the metabolic regulation. Undoubtedly, altered abundance and function of proteins can be considered as a major player in the pathogenesis of many diseases, since most cellular processes are catalyzed by proteins. A deeper characterization of the mitochondrial proteome is strongly needed to better understand the prominent involvement of mitochondrial dysfunction in numerous diseases. Unbiased MS-based proteomics is indeed a potent tool, which enables us to directly investigate and characterize the mitochondrial proteome in several different samples in great depth. However, comprehensive proteomics of complex samples, such as tissues in general and skeletal muscle in particular, is challenging [37]. Below, we will describe emerging developments in proteomic workflows in human muscle mitochondria and also the technical limitations of these strategies.

### Strategies for Identification and Quantification of the Proteome in Human Skeletal Muscle

Global quantitative methods are indispensable for the characterization of the molecular mechanisms and pathways underlying multiple disorders and MS-based proteomics is an ideal approach, since it provides detailed information of the proteome in healthy versus disease states [38,39]. Quantitative proteomics has been undergoing a renaissance recently, which has shifted protein research from studies targeting only a limited number of proteins into a large-scale discovery platform, making it feasible to identify and quantify thousands of proteins simultaneously in an unbiased manner [40,41,42].

The first quantitative proteomic studies of skeletal muscle from individuals with obesity and T2D applied gel-based techniques, such as two-dimensional gel electrophoresis (2-DE) and two-dimensional difference gel electrophoresis (2D-DIGE) [43,44]. In 2-DE, the separation is based on the differences in pI and molecular weight, and even though it provides good coverage of soluble proteins, it is biased against hydrophobic proteins. Another challenge is that the presence of highly abundant proteins, such as structural proteins in skeletal muscle, distorts certain areas of the gel. This will disturb proper identification and quantification of other proteins in the same area. The quantification of protein spots was based on, e.g., Coomassie blue or silver staining combined with software-based image analysis. While Coomassie blue staining has quite a low sensitivity in protein detection, silver staining displays improved sensitivity but may interfere with the subsequent MS-based protein identification [43]. As such, the development of 2-D-DIGE, which includes labeling of proteins with multiplexed fluorescent dyes and separation in the same gel, provides a more accurate quantification [44,45]. Early studies of the mitochondrial proteome in different tissues mainly relied on these gel-based proteomic approaches, but the above-mentioned limitations most likely explain the reduced the number of mitochondrial proteins identified in these studies [46,47]. Due to the challenges in gel-based proteomics, and at the same time, the extensive improvements in the instrumentation and the bioinformatic tools available, MS-based proteomics is nowadays the method of choice for large-scale protein identification and quantification.

**Figure 1:**
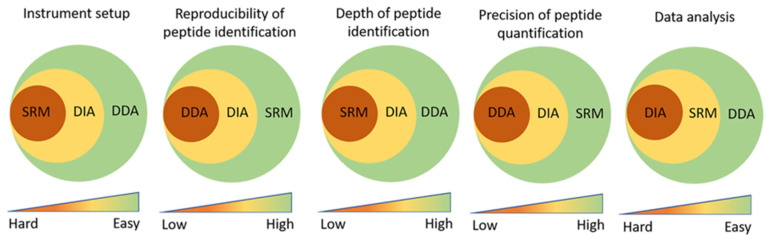
Advantages and limitations of MS-based proteomics using either data-dependent acquisition (DDA), data-independent acquisition (DIA), or the targeted approach called selected reaction monitoring (SRM). SRM requires prior identification of peptides and therefore currently has the hardest instrument setup, while DDA offers the easiest and is the default mode of use on most mass spectrometers. On the other hand, SRM offers the highest reproducibility and is the most sensitive method regarding peptide quantification because of a high signal-to-noise ratio. DIA is almost comparable to SRM with respect to the reproducibility and precision of quantification but is more vulnerable to variation caused by interference from other peptides. While DDA shows the least optimal performance regarding the reproducibility and precision of peptide identification and quantification, it is currently the method of choice for most discovery proteomics studies due to the high depth of peptide identification and easy data analysis.

For most biomedical studies, the global changes in protein abundances or in the level of a particular PTM are of interest. The two main proteomic approaches for such comparative studies are untargeted discovery-oriented proteomics and targeted MS analysis of selected proteins. The untargeted approach can be implemented in two different ways, either by data-dependent analysis (DDA), also known as shotgun proteomics, or by data-independent analysis (DIA), also referred to as SWATH (sequential window acquisition of all theoretical mass spectra). In the DDA approach, the dataset originates from a full spectrum of the peptides at the MS1 level, followed by collection of as many corresponding peptide fragment mass values (MS2) as possible. Using the DDA strategy, there is a risk of not mapping peptides with a very low abundance. However, despite this limitation, DDA is a relatively straightforward strategy in the field of discovery-mode quantitative proteomics [39] (Figure 1).

The data obtained by MS-based proteomics can subsequently be analyzed by different software platforms, such as Maxquant [48] and Perseus environment [49]. Within the DDA approach, both labeled and label-free methods have been developed. Chemical labeling using isobaric tags, such as tandem mass tag (TMT) and isobaric tags for relative and absolute quantitation (iTRAQ), was developed for the relative quantification of protein abundances between samples and they are similar in concept. The isobaric tags are added to the samples after proteolytic digestion, and due to the isobaric nature of the tags, an identical peptide from each sample will appear as a single peak in the MS spectra, whereas the individual tags produce a distinct reporter ion in the MS/MS spectra, thus enabling a relative comparison between samples. Although relative quantification using isobaric tags is accurate, it is an expensive approach and it only enables comparison of a limited number of samples (4- and 8-plex with iTRAQ and 10-plex with TMT). In contrast, label-free quantification enables comparison of an unlimited number of samples; however, it is associated with higher variation since the samples are prepared individually and do not have a labeled internal standard.

In order to increase the probability of mapping the entire proteome, the DIA method has been developed during the last couple of years. This involves fragmentation of all peptide precursor ions within a predefined *m/z* range (e.g., a window of 25 *m/z* units) [50,51]. Subsequently, the multiplexed fragment spectra can be interpreted by a software, such as OpenSWATH [52]. Contrary to the DDA approach, a DIA-based analysis may yield complete coverage of peptides and peptide fragments since it has fewer missing values (Figure 1). It will therefore support both discovery-mode and hypothesis-driven investigations [53], although, the robustness of the analysis relies on the spectral databases [54]. Since SWATH promises a higher sensitivity than targeted proteomics [55] and considering its successful application in recent studies in mice [56,57], SWATH has the potential to become the method of choice for mitochondrial proteomics, especially in combination with mitochondrial proteome databases, such as MitoCarta [58] and Mitominer [59].

Targeted proteomics is a general term for MS-based quantification of a predefined and relatively limited set of proteins or peptides of interest. The method currently comprises two classical approaches, selected reaction monitoring (SRM) and parallel reaction monitoring (PRM). In both these approaches, the mass-to-charge ratio of the precursor ion and the fragment ions, together with the retention times of the targeted peptides, are determined prior to the measurements [60,61]. Subsequently, the quantification is achieved by relating the fragmentation intensities of the targeted peptides to the corresponding signals of isotopically labeled reference peptides using a software, such as Skyline [62]. Even though the targeted approach offers the possibility to address, e.g., 50 to 100 selected proteins in each run as well as more accuracy and precision compared to the classic immunoblotting, there are only a few studies, which have applied a targeted proteomics approach for the characterization of the human mitochondrial proteome. In one study, the authors successfully developed SRM platforms for the quantification of four proteins catalyzing the degradation of branched-chain amino acids in a severe and rare inherited metabolic disorder [63]. Another example is the application of SRM in the molecular characterization of dilated cardiomyopathy, where the authors reported that an increased abundance of peroxiredoxin 3 was associated with impaired ventricular function [64]. They also demonstrated that several mitochondrial proteins, including those encoded by *ODPA*, *DLD*, and *ATP5F1A*, were significantly increased in patients with dilated cardiomyopathy compared with the control group [64]. Wolters et al. also developed an SRM approach to quantify more than 50 mitochondrial proteins in major metabolic pathways in mice, rats, and human samples [65]. Finally, others have applied a targeted SRM approach for identification and quantification of acetylation in mitochondria [66], while an MRM (multiple reaction monitoring) approach has been used for identification and quantification of the mitochondrial phosphoproteome [67,68]. The diverse nature of these studies underlines the great potential of targeted proteomics to expand our knowledge of the proteome and PTMs, such as the acetylome and phosphoproteome, with the ultimate aim of uncovering novel and fundamental modes of metabolic regulation. While the targeted proteomic approach overcomes the limitation of large-scale discovery-mode quantitative proteomics in the sense of detecting and quantifying low-abundance peptides, it will limit the number of proteins that can be quantified from thousands to a hundred, at most. The targeted proteomics approach is also very time consuming and requires prior knowledge of the surrogate peptides and their respective SRM transitions in the sample [69,70]. In comparison, the SWATH approach grants a quantitative assay of complex samples in the same manner as SRM but also enables the monitoring of a higher number of SRM transitions than conventional SRM [69] (Figure 1).

Improved technical abilities at the level of liquid chromatography (LC), such as high-pressure high-performance liquid chromatography (HPLC), the development of fast and sensitive mass spectrometers with high resolution and a greater dynamic range, together with improvements in the protocols for sample preparation and bioinformatics will enable deeper proteomic analyses of skeletal muscle and isolated mitochondria in the future [71,72,73]. As discussed above, there are different quantitative MS-based approaches suitable for mitochondrial proteomics, which all have advantages and disadvantages compared to each other (Figure 1).

## 3. Proteomic Characterization of Human Skeletal Muscle

Despite the tremendous progression of MS-based proteomics over the past two decades, proteomic analyses of skeletal muscle are still challenging. Human skeletal muscle biopsies are highly heterogeneous and often contaminated with connective tissue, blood, and blood vessels. Furthermore, skeletal muscle contains large amounts of structural proteins. This contributes to the wide dynamic range of proteins in skeletal muscle. This major challenge in skeletal muscle proteomics was evident in a study by Deshmukh et al., where the proteomes of mouse skeletal muscle and cultured C2C12 myotubes had a dynamic range that spread over eight orders of magnitude [74]. In mouse muscle, the 10 most highly abundant proteins constituted 50% of the total protein mass, with proteins annotated to the contractile machinery accounting for ~54% of the total protein mass [74]. At the other end of the scale, the lower half of the proteome accounted for less than 0.1% of the total protein mass [74]. These challenges have contributed to the relatively limited depth of skeletal muscle proteome coverage in the studies published so far.

### 3.1. Subcellular Fractionation of Muscle Mitochondria

Subcellular fractionation greatly reduces sample complexity, thus enhancing the number of proteins and/or PTMs identified and quantified in a proteomic study. As the present review focuses on human skeletal muscle mitochondria, the following sections will only focus on the isolation of mitochondria and single muscle fibers. We and others have shown that the use of isolated mitochondria significantly increases the number of peptides and proteins, which can be assigned to mitochondria by MS-based proteomics in human skeletal muscle when compared to studies of whole-cell and whole-tissue extracts [75,76,77]. In two studies using isolated human skeletal muscle mitochondria, Lefort et al. and Zhao et al. assigned 59% and 75% of the peptide tandem MS (MS/MS) spectra to mitochondrial proteins, respectively [76,78], while only 8% of the MS/MS peptide spectra were assigned to mitochondrial proteins in a proteomic characterization of whole-cell lysates from human skeletal muscle [77].

It is often a challenge to obtain reliable and reproducible data when performing proteomic studies of isolated organelles, including isolated mitochondria. The protocols used for purification of mitochondria are obviously crucial as all mitochondrial isolation procedures will result in contamination with non-mitochondrial proteins. Therefore, the mitochondrial purity and functionality should be evaluated, for instance, by Western blotting [76,79], assessment of respiratory measurements [76,78,80,81], membrane potential measurements [81], cytochrome C oxidase assay [80], and/or electron microscopy for morphological examination [79]. Additionally, the fact that skeletal muscle contains two distinct mitochondrial subpopulations, the subsarcolemmal (SS) and the intermyofibrillar (IMF) mitochondria, should be considered. Several studies have suggested that the two mitochondrial subpopulations differ both functionally and morphologically [82,83,84,85,86]. Of particular interest, a reduced function and content of SS mitochondria have been reported in skeletal muscle of patients with T2D compared with matched obese individuals [15]. Although the isolation procedure requires relatively large tissue samples and may cause some disruption of the mitochondrial structure and morphology [80,87], the proteomic characterization of isolated mitochondria could be a useful approach to enhance the number of proteins and/or PTMs identified and quantified in a proteomic study.

### 3.2. Proteomic Characterization of Isolated Single Fibers

Adult human skeletal muscle consists of three major fiber types: Slow oxidative type 1 fibers, fast oxidative type 2a fibers, and fast glycolytic type 2x fibers [88,89]. These muscle fiber types differ metabolically, thus contributing to the heterogeneity of muscle samples. The mixed fiber-type composition of most mammalian skeletal muscles therefore complicates the interpretation of data obtained on whole-tissue samples as they reflect the fiber-type composition of the muscle biopsy [90]. The fiber-type composition differs from one type of muscle to another [91] and may be remodeled by hormonal and metabolic changes as well as by muscle activity [92,93]. So far, most proteomic studies of human skeletal muscle have been performed on the vastus lateralis muscle [75,76,77,78,94,95,96,97,98,99,100,101,102,103], which is a mixed-fiber muscle. However, since MS-based studies have revealed differences between the proteomes of human vastus lateralis, trapezius, and deltoideus muscle [104,105], findings should be extended from one group of muscle to another with caution.

The heterogeneity arising from a mixed fiber-type composition may be circumvented by studying isolated single muscle fibers. Characterization of isolated single fibers from biceps muscle biopsies in healthy humans revealed metabolic heterogeneity between the different muscle fibers as there appears to be a higher prevalence of glycolytic enzymes in type 2 fibers and an increased content of mitochondrial oxidative enzymes in type 1 fibers [106]. These findings were supported by a comprehensive study by Murgia et al., in which they characterized the proteome of single muscle fibers from the extensor digitorum longus (EDL) muscle in mice using an MS-based proteomics approach [107]. In this study, more than 5400 proteins were identified in 152 single muscle fibers, with fast type 2x fibers showing a higher abundance of components from the sarcoplasmic reticulum and the T-tubule system [107]. The abundance of proteins involved in OXPHOS, β-oxidation, and the TCA cycle varied significantly between the individual muscle fiber types, with type 2a fibers and type 1 fibers showing a higher abundance of OXPHOS proteins and proteins involved in β-oxidation, respectively [107]. The impressive number of skeletal muscle proteins identified by Murgia et al. clearly underlines the fact that single fiber subfractionation can increase proteome coverage substantially [108]. Furthermore, a study of skeletal muscle biopsies from patients with T2D and matched healthy lean and obese individuals suggested that human type 1 muscle fibers have a higher capacity for glucose handling, but a similar insulin-mediated regulation of protein phosphorylation [109]. Thus, Albers et al. found impaired insulin-stimulated phosphorylation of Akt at Ser473 and Thr308 in muscle from patients with T2D, but with a similar reduction in type 1 and 2 fibers [109]. This study only characterized the protein abundance and the phosphorylation of relatively few proteins of interest using Western blotting, whereas an unbiased quantitative MS-based approach most likely would have revealed multiple novel fiber-type-specific differences in protein abundance and phosphorylation between the study groups. Although proteomic studies of isolated single muscle fibers may provide a more homogenous sample and thus allow a more accurate characterization of the skeletal muscle proteome, the small protein amount available in isolated fibers will make it challenging to obtain in-depth characterization of low-abundance proteins and PTMs.

## 4. Biological Aspects of the Mitochondrial Proteome of Human Skeletal Muscle

The mammalian mitochondrial proteome has been estimated to range from 1000 to 4200 proteins [79,110,111], with the mitochondrial proteome of humans being estimated to contain approximately 1500 proteins, although the number varies between tissues and cell types [110,112]. Recently, The Human Skeletal Muscle Proteome Project was initiated, which aims to characterize the human skeletal muscle proteome. As a part of this initiative, Gonzalez-Freire et al. compiled findings from 38 publications published from 2002 to 2015, which led to the identification of 5431 non-redundant proteins in human skeletal muscle [113]. From this list, 5031 proteins were assigned to structural compartments, including cytoplasm (2302 proteins), cell membrane (1870 proteins), nucleus (1479 proteins), and mitochondria (798 proteins), thus suggesting that only half of the estimated mitochondrial proteome in human skeletal muscle was covered at this time point. In the following sections, we will summarize the results of proteomic studies that have led to the identification of the current mitochondrial proteome in skeletal muscle of healthy humans as well as the changes observed in obesity, T2D, and aging, which are all characterized by IR. We will furthermore describe the proteomic studies that have aimed to characterize the effect of exercise on the human skeletal muscle proteome.

### 4.1. Discovery-Mode Proteomics of Whole Muscle and Isolated Mitochondria in Humans

**Figure 2:**
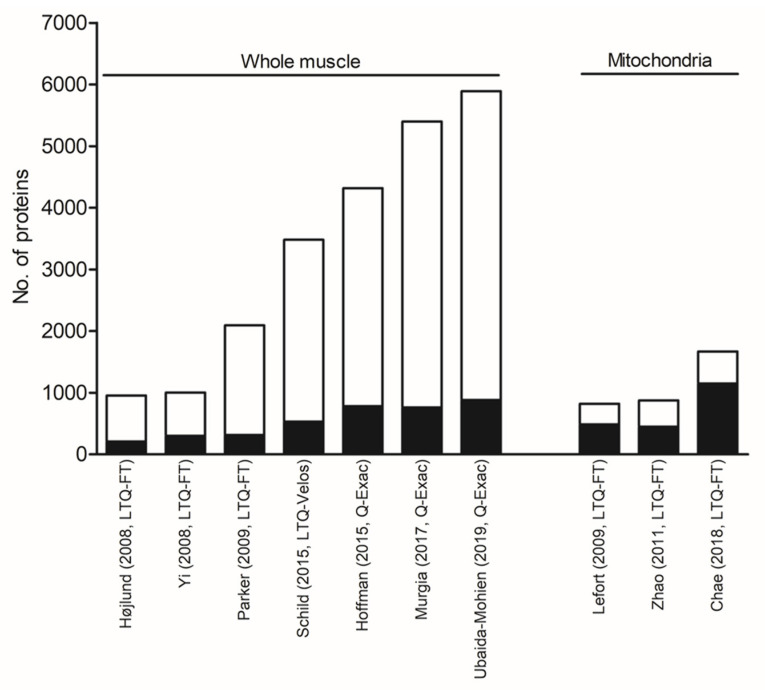
The number of proteins identified using discovery-mode proteomics on whole-muscle samples and isolated mitochondria from human skeletal muscle. The black part of the bars shows the number of non-mitochondrial proteins, while the white part represents the number of mitochondrial proteins identified in the respective studies identified by the first author, year of publication, and instrumental setup.

Several studies have aimed to characterize the proteome of human skeletal muscle. For a complete overview of studies published until 2015, we refer to the review by Gonzalez-Freire et al. [113]. Here, we briefly describe studies, which to a major extent have contributed to the proteomic characterization of whole-muscle and muscle mitochondria in humans (Figure 2).

More than a decade ago, Højlund et al. took advantage of one-dimensional (1-DE) gel electrophoresis and HPLC-ESI-MS/MS to characterize the proteome of human vastus lateralis muscle from three healthy individuals [77]. Of the 954 unique proteins identified, 22% were assigned to mitochondria using the Gene Ontology annotation information in the UniProt database (Figure 2). Among the proteins assigned to mitochondria were 55 out of 88 subunits in the OXPHOS machinery but also several carrier and transfer proteins, the majority of proteins involved in transport activation and the transfer of fatty acids into mitochondria, and the subsequent degradation of fatty acids through β-oxidation [77]. In a similar study using similar technologies [114], Yi et al. identified 1003 unique proteins in skeletal muscle biopsies from six healthy human volunteers, of which 30% were reported to be assigned to mitochondria (Figure 2). In a concurrent study by Parker et al., they demonstrated that using a higher number of muscle biopsies markedly increases the number of proteins identified. They took advantage of four different workflows on 31 muscle biopsy samples from patients with various muscle diseases, resulting in 178 4h HPLC runs [115]. This comprehensive effort led to the identification of 2095 proteins (Figure 2), of which 315 proteins (15%) were assigned to mitochondria according to Gonzalez-Freire et al. [113,115].

More recently, the improved technical and analytical tools used in MS-based proteomics today have greatly increased the number of proteins identified. Thus, Schild et al. characterized the skeletal muscle proteome of endurance-trained individuals versus untrained individuals using a label-free proteomics approach in which the whole-muscle lysates were analyzed using off-gel HPLC-MS/MS [97]. This study led to the identification of 3481 unique proteins of which 533 proteins (15%) could be assigned to a mitochondria-related “cellular-component” GO-term (Figure 2). Furthermore, Hoffman et al. took advantage of nano-ultra HPLC-MS/MS to identify proteins in a study and characterized the effect of acute exercise in whole-muscle lysates from healthy individuals [102]. This led to the identification of 4317 muscle proteins of which 781 (18%) were assigned to mitochondria by Gonzalez-Freire et al. [102,113]. In very recent studies of aging by Murgia et al. [108] and Ubaida-Mohien et al. [116], the proteomic coverage of human skeletal muscle and proteins assigned to mitochondria were even further increased as described below (see also Figure 2).

In 2009, Lefort et al. carried out the first characterization of the mitochondrial proteome using isolated functional mitochondria from human skeletal muscle [76]. Using 1-DE and HPLC-ESI-MS/MS, this study led to the identification of 823 unique proteins of which 487 proteins (59%) were assigned to mitochondria (Figure 2). Of the 487 mitochondrial proteins identified by Lefort et al. [76], only 54% were detected using the same MS-based proteomic approach on whole-tissue homogenates by Hojlund et al. [77], thus emphasizing the advantages of subcellular fractionation. This was further underlined in a study by Zhao et al., where isolated mitochondria from healthy human skeletal muscle were subjected to 1-DE for separation, in-gel digestion, and LC-MS/MS for analysis of peptides in order to characterize the mitochondrial proteome and phosphoproteome [78]. In this study, 878 unique proteins were identified of which 448 proteins (51%) were assigned to mitochondria (Figure 2).

In a more recent study [117], Chae et al. published the, so far, most comprehensive mitochondrial proteome of human skeletal muscle. Chae and co-authors implemented ultra-high-pressure nano-LC-ESI-MS/MS on isolated mitochondria from skeletal muscle of T2D patients and non-diabetic control individuals and identified 1671 proteins in at least half of the 10 samples [117]. Of these proteins, 1150 mitochondrial proteins (69%) were represented by two or more peptides (Figure 2). This study clearly underlines how the improved technical and analytical tools in MS-based proteomics, in particular in combination with the use of isolated mitochondria, have the potential to enable an even deeper characterization of the mitochondrial proteome in human skeletal muscle in the near future.

Taken together, these studies clearly show that the significant advances in MS-based proteomics within the past decades have markedly increased the proteomic coverage of human skeletal muscle and mitochondrial proteins, and that subcellular fractionation by the use of isolated mitochondria greatly improves the relative fraction of mitochondrial proteins identified (Figure 2). However, a relatively large proportion of the proteins identified in isolated mitochondria were not assigned to mitochondria. It could be of great interest to further characterize these proteins to determine whether they arise from non-mitochondrial contamination or cellular organelles known to interact with mitochondria [118] and whether they play a role in mitochondrial metabolism. The latter will possibly enable us to gain novel information about the processes occurring in human skeletal muscle mitochondria.

### 4.2. Discovery-Mode PTM Proteomics of Whole Muscle and Isolated Mitochondria in Humans

One of the most prevalent PTM of proteins is reversible phosphorylation, which has emerged as an essential regulator of metabolic processes, also in mitochondria [119,120]. As was the case when characterizing the mitochondrial proteome in human skeletal muscle, characterization of the phosphoproteome greatly benefits from mitochondrial enrichment prior to the mass spectrometry experiments. Using whole-muscle samples, we identified 306 unique phosphorylation sites in 127 proteins when taking advantage of strong cation exchange (SCX), TiO_2_-mediated phosphopeptide enrichment, and HPLC-ESI-MS/MS. Of these, 31 phosphoproteins (22%) were assigned to mitochondria [75]. Lundby et al. identified 3372 phosphopeptides in 1532 phosphoproteins in human skeletal muscle [121], of which 129 proteins were assigned to mitochondria according to Gonzalez-Freire et al. [113,121]. In comparison, Zhao et al. combined mitochondrial isolation with TiO_2_-mediated phosphopeptide enrichment and LC-MS/MS and were able to identify 155 distinct phosphorylation sites in 77 mitochondrial phosphoproteins [78]. These phosphoproteins sites were involved in numerous mitochondrial processes, including OXPHOS, the TCA cycle, lipid metabolism, amino acid degradation, import, transport, calcium homeostasis, and apoptosis [78]. In a subsequent study, Zhao et al. investigated the effect of a 4h insulin infusion on the phosphorylation of mitochondrial proteins in isolated muscle mitochondria from healthy individuals by taking advantage of TiO_2_-mediated phosphopeptide enrichment, hydrophilic interaction liquid chromatography (HILIC) fractionation, and LC-MS/MS [95]. A total of 207 phosphorylation sites were identified and assigned to 95 mitochondrial phosphoproteins, of which 45% were identified in both the basal- and insulin-stimulated state. The identified proteins were assigned to mitochondrial processes, such as OXPHOS, the TCA cycle, lipid metabolism, the mitochondrial inner membrane organizing system (MINOS), calcium homeostasis, etc. [95]. Interestingly, insulin caused a two-fold increase in the number of mitochondrial phosphorylation sites and phosphoproteins identified, and the phosphorylation sites that were identified only in the insulin-stimulated state included proteins involved in OXPHOS, the TCA cycle, and the import machinery/transporters, thus providing evidence that insulin modulates mitochondrial metabolism through protein phosphorylation.

A recent study by Hoffman et al. characterized the effect of exercise on protein phosphorylation in whole-muscle lysates [102]. This study took advantage of isobaric labeling for quantification, TiO_2_ and sequential elution from IMAC (SIMAC) for phosphopeptide enrichment, HILIC fractionation, and nano-ultra HPLC coupled to tandem MS for analysis. In total, 11903 phosphosites and 4317 proteins were identified in skeletal muscle from 4 healthy individuals [102]. The number of proteins and phosphorylation sites identified in the study by Hoffman et al. is promising for future proteomic studies aiming to characterize the skeletal muscle proteome or the mitochondrial proteome in both healthy individuals as well as in response to physiological interventions and various diseases.

Another PTM that has gained increased attention during the last decade is lysine acetylation as it is evolutionarily conserved and has been shown to regulate the function of several proteins [122,123,124,125]. Mielke et al. characterized the acetylome in isolated muscle mitochondria from healthy lean and obese non-diabetic volunteers [125]. Interestingly, Mielke et al. found that the number of spectra assigned to acetylated peptides from mitochondrial proteins and normalized to the total number of spectra for mitochondrial proteins using the normalized spectral abundance factor (NSAF) approach correlated positively with whole-body insulin sensitivity [125]. This study identified ADP/ATP translocase 1 (ANT1), a translocase that shuttles ADP and ATP across the inner mitochondrial membrane, as being consistently acetylated on lysine 10, 23, and 92 [125]. The side chain of lysine residues contributes to the positive charge of ANT1 that is critical for the transport of ADP and ATP and molecular modeling showed that acetylation of Lys23 profoundly reduces the affinity of ADP for ANT1, thus also influencing the mitochondrial ATP production rate [125,126]. The study by Mielke et al. was carried out on whole-muscle lysates, and we therefore anticipate that enrichment of acetylated peptides using immunoprecipitation will greatly enhance the number of mitochondrial acetylation sites detected and thus the information gained from a proteomic experiment.

Combined, the abovementioned studies have sought to characterize the skeletal muscle phosphoproteome and acetylome of healthy human individuals, which may serve as an inspiration for similar studies in various interventions and disease models as described in the following sections. Particularly, the large number of phosphorylation sites identified on whole-muscle samples by Hoffman et al. is promising for any future attempts to characterize the mitochondrial phosphoproteome or acetylome. In particular, it will be interesting to exploit the technical and analytical improvements to further characterize how post-translational modifications of mitochondrial proteins are regulated under different metabolic conditions, e.g., in response to insulin stimulation or exercise, as well as in individuals with obesity, T2D, and aging.

### 4.3. Altered Mitochondrial Proteomes in Obesity and Type 2 Diabetes

**Figure 3:**
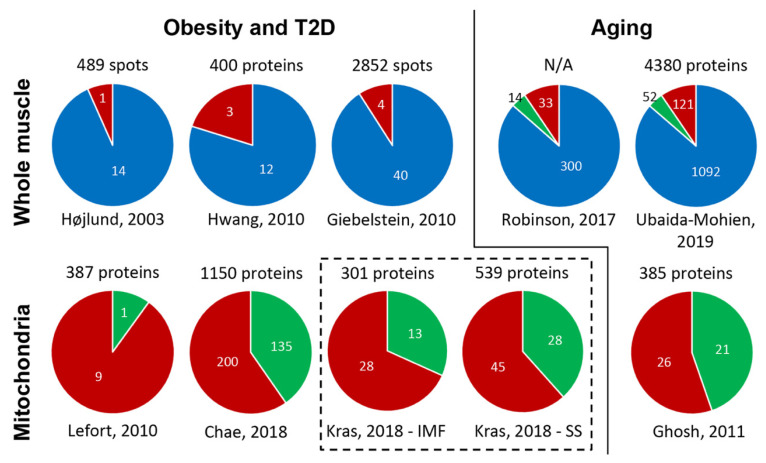
Altered mitochondrial proteome in skeletal muscle in obesity, T2D, and aging. The pie charts show the total number of protein spots or proteins quantified in whole muscle or the total number of mitochondrial proteins quantified in isolated mitochondria (top), and the number of regulated non-mitochondrial (blue) proteins and downregulated (red) or upregulated (green) mitochondrial proteins identified in relation to obesity, T2D, or aging in proteomic studies of human skeletal muscle (whole muscle or isolated mitochondria) published (first author, year of publication). The pie charts in the box with dotted lines represent a study of both intermyofibrillar (IMF) and subsarcolemmal (SS) muscle mitochondria. N/A, not available.

IR in skeletal muscle of patients with T2D plays a major role in the development of disease. While the molecular mechanisms underlying IR in obesity and T2D remain to be established, multiple studies have provided evidence for a link between mitochondrial dysfunction and IR in human skeletal muscle [5,127,128,129]. For these reasons, there has been a steady interest in the application of proteomic approaches for studying the mitochondrial proteome in human skeletal muscle biopsies or mouse models. In the present section, we review the results of proteomics-based investigations of human skeletal muscle biopsies from individuals with obesity and T2D (Figure 3).

The identity of the proteins mentioned below is given by either the name of protein and/or the gene name encoding them.

In 2003, our group applied a proteomics approach involving 2-DE, image analysis, and MALDI-TOF MS for the identification of changes in the skeletal muscle proteome of patients with T2D [94]. The study showed an altered abundance of 15 protein spots, of which 11 protein spots were identified (8 unique proteins) in the skeletal muscle of patients with T2D compared with healthy controls, and hence possibly related to altered metabolism in T2D (Figure 3). We found a reduced abundance and altered phosphorylation of the mitochondrial protein ATP synthase β-subunit (encoded by *ATP5B*) in the skeletal muscle of patients with T2D, and also identified a phosphorylation site at Thr213 in the nucleotide-binding region of the catalytic β-subunit of the ATP synthase complex [94]. In a follow-up study, we immunoprecipitated ATP synthase β-subunit from human skeletal muscle biopsies and applied HPLC-MS/MS to identify seven phosphorylation sites in the ATP synthase β-subunit, of which two (Thr213 and Tyr361) were increased in obesity and T2D [130]. In addition to altered phosphorylation of the ATP synthase β-subunit, we demonstrated a reduced protein abundance of several subunits in the mitochondrial respiratory complexes using Western blotting, thus supporting a link between mitochondrial dysfunction and IR in skeletal muscle in obesity and T2D [130].

In 2010, Hwang et al. carried out another comprehensive proteomics-based study of whole-muscle lysates from patients with T2D and lean and obese non-diabetic individuals [99]. Using 1-DE, HPLC-ESI MS/MS, and the NSAF method for quantification, this study identified 1218 proteins in at least one of the study participants, while 400 proteins were assigned in at least half of the individuals (Figure 3). Fifteen proteins were identified as significantly altered, of which there was a reduced abundance of mitochondrial proteins, including cytochrome C oxidase VIC precursor (*COX6C*), coiled-coil-helix-coiled-coil-helix domain-containing protein 3 (*CHCHD3*), and ubiquinol-cytochrome C reductase complex ubiquinone-binding protein QP-C (*UQCRQ*), in obese and T2D individuals [99]. Overall, the study revealed patterns of decreased abundance of mitochondrial proteins in skeletal muscle in obesity and T2D [99].

The study by Giebelstein et al. provided yet another example of how proteomics can contribute to the understanding of the molecular mechanisms underlying IR in skeletal muscle [101]. In this study, Giebelstein et al. used 2-D-DIGE for separation and protein quantification and nano-HPLC/ESI-MS/MS for protein identification to compare the skeletal muscle proteome of patients with T2D with that of healthy lean and obese non-diabetic individuals [101]. A total of 44 protein spots (26 unique proteins) were found to be significantly altered in patients with T2D and/or obesity versus lean individuals in either the basal state or after insulin administration. Giebelstein et al. [101] found a higher abundance of glycolytic proteins, such as enzymes encoded by *GAPDH*, *PGAM2*, *ENO3*, and *PKM2*, in obese individuals with and without T2D, while mitochondrial proteins (encoded by *ECH1*, *GBAS*, *HADHB*, and *HES1*) were downregulated in skeletal muscle from these insulin-resistant individuals (Figure 3). Interestingly, these changes were associated with a shift in markers of muscle properties, since fast muscle proteins, such as myosin light chain (*MYL*) 1, myosin regulatory light chain 2 (*MYLPF*), and fast skeletal muscle troponin-I (*TNNT3*), were upregulated, whereas slow muscle proteins, such as other myosin light chain isoforms (*MYL2*, *MYL3*) and slow skeletal muscle troponin-T (*TNNT1*), were downregulated in obesity and T2D [101].

While Hwang et al. analyzed whole-muscle lysates [99], Lefort et al. applied 1-DE and HPLC-ESI-MS/MS on isolated muscle mitochondria to compare the abundance of mitochondrial proteins in skeletal muscle mitochondria from lean insulin-sensitive and obese insulin-resistant individuals [100]. This study identified a total of 691 proteins, of which 10 mitochondrial proteins showed an altered abundance in insulin-resistant obese individuals (Figure 3). Interestingly, the abundance of several complex I subunits of the NADH dehydrogenase (ubiquinone) family and a subunit in complex IV, cytochrome c oxidase subunit Vb (*COX5B*) was reduced in obese individuals, while the protein content of subunits in the respiratory complexes II and III was unchanged [100]. The lower abundance of complex I subunits may have contributed to the increased ROS production reported in these insulin-resistant obese individuals [100]. However, peroxiredoxin-2 (*PRDX2*), a protein that protects against oxidative stress, was upregulated in obese individuals. In line with the strong association between circulating levels of branched-chain amino acids and IR [131,132,133,134] and the intramyocellular accumulation of lipids in muscle from individuals with IR [135], the authors found lower levels of carnitine palmitoyltransferase 1β and enzymes involved in branched-chain amino acid metabolism, such as those encoded by *ALDH6A1* and *PCCB*, in insulin-resistant obese individuals [100].

The technical advances that have occurred in recent years within the field of MS-based proteomics are particularly evident in the latest attempt to characterize the mitochondrial proteome in skeletal muscle of patients with T2D. As mentioned above, Chae and co-authors used HPLC-ESI-MS/MS on isolated mitochondria from skeletal muscle of patients with T2D and non-diabetic controls and identified a total of 1150 mitochondrial proteins that were represented by two or more peptides and which were therefore selected for subsequent analysis [117]. Among the 335 differentially regulated mitochondrial proteins, 135 proteins were upregulated in T2D compared with the nondiabetic controls, whereas 200 proteins were downregulated (Figure 3). The proteins upregulated in patients with T2D were mainly involved in processes related to molecular transport and cytoskeletal organization, while Chae et al. showed a significant downregulation of several proteins in metabolic pathways, including the TCA cycle, fatty acid oxidation, and glycolysis, and in T2D patients [117]. Interestingly, OXPHOS proteins were represented both among the upregulated and downregulated proteins (6 and 16 proteins, respectively), suggesting an overall altered metabolic activity in patients with T2D, when compared to healthy individuals.

As stated above, skeletal muscle contains two distinct mitochondrial subpopulations, the SS and the IMF mitochondria. In a recent comprehensive study, Kras et al. used label-free quantitative tandem mass spectrometry (HPLC-ESI-MS/MS) and spectral counting (NASF) to evaluate the abundance of mitochondrial proteins in two distinct subpopulations of SS and IMF mitochondria obtained from skeletal muscle of lean individuals and obese individuals [136]. Using only proteins identified as mitochondrial by GO:0005739, a total of 539 and 301 mitochondrial proteins were identified in isolated SS and IMF mitochondria fractions, respectively, in more than half of the 33 study participants [136]. Of these, 73 and 41 mitochondrial proteins showed an altered abundance in SS and IMF mitochondrial fractions, respectively, in obese individuals (Figure 3). Interestingly, the protein content of subunits in the respiratory complexes I, III, and V (ATP synthase) were lower, while the abundance of certain TCA cycle proteins and complex II proteins were higher in muscle IMF mitochondria from obese individuals [136]. Obesity therefore appears to be associated with differential effects on the mitochondrial oxidative metabolism in a mitochondrial subpopulation-dependent manner.

Combined, the proteomics studies presented in this section show that the mitochondrial proteome is altered in skeletal muscle of individuals with obesity and T2D, most often with the majority of regulated mitochondrial proteins being downregulated, but with the recent improvements in the proteomic coverage of muscle mitochondrial proteins also demonstrating that a fraction of mitochondrial proteins are upregulated (Figure 3). While these results have provided further insight into the observed link between mitochondrial dysfunction and IR in skeletal muscle in obesity and T2D, the full proteomic signature of this association remains to be established. As stated in the previous section, the advances made in MS-based proteomics and the improved protocols for subfractionation already now enable us to gain an even deeper understanding of the mitochondrial proteome and how this is influenced by obesity and T2D.

### 4.4. Altered Mitochondrial Proteome with Aging

Aging is associated with numerous mitochondrial alterations, including reduced mitochondrial function and content and a decreased synthesis of mitochondrial proteins [17,137,138,139,140,141]. Aging is characterized by an impaired glucose tolerance, and mitochondrial dysfunction in skeletal muscle has been suggested as being implicated in the development of IR with age [17,142]. Characterization of the changes that occur in the mitochondrial proteome with age could therefore potentially give an insight into the concurrent changes in metabolic function.

Robinson et al. used a DDA label-free proteomic approach and nano LC-ESI-MS/MS on whole-muscle homogenates to identify a reduced abundance of 127 proteins and an increased abundance of 220 proteins in the skeletal muscle of older (age 65–80 years) compared to younger individuals (age 18–30 years) [143]. This included 33 mitochondrial proteins with a reduced abundance and 14 mitochondrial proteins with an increased abundance in the skeletal muscle of older individuals (Figure 3). These findings were consistent with a decrease in the mitochondrial respiratory capacity in the older group at baseline [143]. In a more recent comprehensive study, Ubaida-Mohien et al. collected skeletal muscle biopsies from 60 healthy individuals aged 20 to 87 years old, divided them into age strata, and used tandem mass tag (TMT) labeling in combination with tandem shotgun MS-based quantitative proteomics to characterize age-related differences in the skeletal muscle proteome [116]. A total of 4380 proteins were quantified in at least 15 participants. Of these, 1265 showed significant age-related changes in protein abundance [116]. As shown previously, proteins implicated in mitochondrial metabolism, but also muscle contraction, muscle architecture, and ribosome function, were decreased with older age, whereas transcriptional regulators and proteins related to splicing, neuromuscular junctions, and senescence were increased with increasing age [116]. Interestingly, of the 884 mitochondrial proteins (15%) quantified, the abundance of 173 proteins changed with age, of which 70% showed a reduced abundance with age (Figure 3). Among the age-related downregulated mitochondrial proteins, 16 proteins were components in the electron transport chain or TCA cycle enzymes, such as malate dehydrogenase, isocitrate dehydrogenase, fumarate hydratase, and succinate-CoA ligase [116]. These findings extend those identified by others and underline the fact that mitochondrial metabolism is indeed altered in aging skeletal muscle.

Ghosh et al. took advantage of isolated mitochondria from skeletal muscle, 1-DE for protein separation, HPLC-ESI-MS/MS for protein assignment, and the NSAF method for quantification and were able to identify 385 mitochondrial proteins, of which the abundance of 47 proteins differed significantly between younger (age 18–30 years) and older individuals (age ≥ 65 years) [137]. Most notably, the abundance of eight subunits in complex I as well as five other OXPHOS subunits was reduced in the older individuals, thus supporting the finding of reduced ATP synthesis in the muscle of older individuals (Figure 3). Additionally, the older individuals had a reduced abundance of PGC-1α mRNA expression, and reduced protein content of nuclear respiratory factor 1 and mitochondrial transcription factor A, which may help to explain the mitochondrial dysfunction observed with aging in skeletal muscle [137].

In a comprehensive study, Murgia et al. characterized the age-related changes in skeletal muscle in a fiber-type-specific manner [108]. They took advantage of a DDA-based LC-MS/MS analysis of isolated slow oxidative type 1 fibers and fast oxidative type 2a fibers from skeletal muscle biopsies of four younger (age 22–27 years) and four older (age 65–75 years) healthy individuals. Immunohistochemical staining of the muscle biopsies revealed a reduced fiber size of the fast type 2a fibers in aging muscle, while there was no change in the size of slow type 1 fibers [108]. As stated previously, Murgia et al. identified more than 5400 proteins in 152 single muscle fibers, which included 759 mitochondrial proteins (14%) [108]. The proteomic characterization revealed that proteins related to OXPHOS are more abundant in type 1 than type 2a fibers and that a reduced abundance of OXPHOS proteins was identified with aging, irrespective of the fiber type. In line with the reduced fiber size of fast type 2a muscle fibers with age, the proteomic analysis identified a reduced abundance of proteins involved in mitochondrial fusion (*MFN2* and *OPA1*), in particular, in type 2a muscle fibers from older individuals [108], whereas the abundance of proteins involved in autophagy (encoded by *SQSTM1*, *GABARAPL2*, and *BNIP3*) and mitochondrial fission (encoded by *DNM2*) was increased in older compared with younger individuals. These results suggest that a switch in the mitochondrial dynamics towards fission and mitophagy contributes to the decline in mitochondrial content and perhaps the reduced size of fast type 2a fibers with age [108].

In summary, the above-mentioned proteomics studies consistently show that the abundance of the majority of mitochondrial proteins is reduced with aging, but as in obesity and T2D, some are also upregulated (Figure 3). These data provide several novel processes in mitochondria of interest, which should be further studied to understand the role of mitochondrial dysfunction in aging muscle and its relation to IR.

### 4.5. Effect of Exercise on the Muscle Mitochondrial Proteome in Healthy Individuals

Endurance exercise training is known to increase whole-body maximal oxygen consumption and insulin sensitivity in humans [93,144]. On a molecular level, the adaptions to exercise in human skeletal muscle include an increased abundance of proteins involved in insulin signaling and glucose metabolism [26,145], while also increasing the mitochondrial content and quality through the induction of mitochondrial biogenesis and dynamics [28,146,147,148,149]. Holloway et al. were the first to characterize the effects of endurance training (six weeks of interval exercise training with three sessions per week) on the human skeletal muscle proteome in five recreationally active men [98]. As expected, interval training increased VO_2_ max, which was concurrent with significantly increased expression of 20 protein spots. Protein separation using 2-DE combined with image analysis and protein identification using LC-MALDI-MS/MS revealed that the abundance of the mitochondrial proteins ATP synthase subunit α and β, NADH dehydrogenase, and succinate dehydrogenase were increased after the training intervention. Interestingly, for the majority of gene products identified, several gel spots were detected, which may indicate regulation through various PTMs in response to exercise training.

Schild et al. characterized the skeletal muscle proteome of endurance-trained individuals versus untrained individuals using a label-free proteomics approach in which the whole-muscle lysates were analyzed using off-gel LC-MS/MS [97]. Schild et al. identified 3481 unique proteins, of which 702 proteins could be identified and quantified in all samples (*n* = 5 in each group) [97]. A total of 92 proteins differed significantly between endurance-trained and untrained individuals, with 70 proteins showing a higher abundance and 22 proteins showing a lower abundance in the endurance-trained group. Almost half of the 92 differentially regulated proteins were assigned to the mitochondrial proteome by GO annotation and the abundance of the majority of these proteins was increased by endurance training [97]. This included proteins involved in OXPHOS, enzymes related to the TCA cycle, fatty acid utilization, the malate-aspartate shuttle, and branched-chain amino acid metabolism [97], thus supporting the previous findings of improved mitochondrial metabolism in response to endurance exercise.

In a study of isolated muscle mitochondria from healthy males undergoing endurance training every day for 14 consecutive days, Egan et al. used 2-D-DIGE analysis with subsequent LC-MS/MS analysis to characterize the effects of training on the mitochondrial proteome [96]. Compared with muscle biopsies taken at baseline, 31 protein spots showed a differential abundance in biopsies obtained after either 7 or 14 days of training [96]. The proteins showing an increased abundance following training included OXPHOS proteins, enzymes in the TCA cycle, and regulators of mitochondrial protein synthesis, thus demonstrating that 7 days of training is enough to promote changes in the mitochondrial proteome of skeletal muscle [96]. These findings were confirmed by Western blotting and qRT-PCR in a subsequent study of the same muscle biopsies, which also found that the gene expression and protein content of PGC-1α and estrogen-related receptor alpha mRNA were induced from day 3 onwards [150]. The increase in these transcription factors known to be mediators of mitochondrial biogenesis may explain the observed increase in mitochondrial proteins observed after 7 and 14 days of endurance training.

As mentioned above, Hoffman et al. characterized the effect of exercise on protein phosphorylation in whole-muscle lysates using advanced MS-based phosphoproteomics [102]. In total, 1322 phosphopeptides were significantly regulated by a single bout of high-intensity exercise at 85%, increasing to 92% of the maximal watts produced (Wmax) for 9–11 min, while only five proteins showed an altered abundance following the acute-exercise intervention [102], suggesting that the immediate response to exercise is mediated through changes in the PTM pattern rather than by changes in protein abundance.

A recent systematic review and meta-analysis found that the abundance of only three proteins (encoded by *NDUFAB*, *NDUFA13*, and *ATPB*) have been reported to be significantly regulated by exercise training in more than one of the studies, which investigated the exercise-mediated changes in the skeletal muscle proteome of healthy individuals [151]. This underlines the complexity of carrying out quantitative proteomic studies of human skeletal muscle as the lack of consistency may reflect the use of different proteomic approaches for protein separation, identification, and quantification, but perhaps even more important is also the fact that the response to exercise differs tremendously depending on the exercise regime and the cohorts being investigated.

### 4.6. Effect of Exercise on the Muscle Mitochondrial Proteome in T2D and Aging

To our knowledge, only one study has investigated the effect of endurance exercise training on the skeletal muscle proteome in patients with T2D. Hussey et al. used 1-DE, HPLC-ESI MS/MS, and the NSAF method for quantification and reported that exercise training altered the abundance of 17 muscle proteins, of which 12 proteins showed an increased and 5 a decreased abundance [103]. The muscle proteins showing an exercise-mediated increase in abundance included several mitochondrial proteins, such as creatine kinase (*CKMT2*), aspartate aminotransferase (*GOT2*), acetyl-CoA acetyltransferase (*ACAT1*), cytochrome b-c1 complex subunit 2 (*UQCRC2*), isoform 1 of succinyl-CoA ligase subunit beta (*SUCLA2*), fumarase (*FH*), trifunctional enzyme subunit alpha (*HADHA*), and isocitrate dehydrogenase (*IDH2*) [103]. Given the improved technical and bioinformatic abilities of the current available proteomic approaches, future studies of the effect of exercise training on the skeletal muscle proteome in T2D patients may elucidate whether exercise training elicits similar beneficial adaptions in the skeletal muscle of patients with T2D as in healthy individuals.

To evaluate the long-term effect of exercise training on mitochondrial health, Lanza et al. recruited younger (age 18–30 years) and older (age 59–76 years) sedentary and endurance-trained individuals to characterize their muscle function and muscle proteome. In general, age was associated with a decreased muscle mass while insulin sensitivity was not influenced by age per se. However, insulin sensitivity was significantly higher in trained individuals compared with sedentary people independent of age [152]. Likewise, the skeletal muscle ATP production rates, citrate synthase activity, and mtDNA copy number were reduced in older individuals but were higher in endurance-trained individuals compared with sedentary people [152]. Consistent with improved muscle ATP production and an increased mtDNA abundance in the older endurance-trained population, a proteomic characterization, using iTRAQ labeling followed by LC-MS/MS, showed that although 27 oxidative and glycolytic proteins were lower in the older sedentary group when compared to the younger sedentary group, there was, with the exception of the three cytochrome c subunits and an aminotransferase enzyme, no apparent differences in protein abundance between the older endurance-trained group and the younger counterpart. These data suggest that the effect of long-term endurance exercise may postpone the age-related decline in mitochondrial mass and function [152].

As described in the previous section, Ghosh et al. compared the proteome of isolated mitochondria from younger (age 18–30 years) and older (age ≥ 65 years) subjects by taking advantage of isolated mitochondria from skeletal muscle and label-free MS/MS-based proteomics for quantification [137]. Interestingly, a 16-week supervised aerobic exercise intervention on stationary bikes increased the insulin sensitivity, VO_2_ max, and ATP production among both the older and younger individuals [137]. Furthermore, the age-related differences in mitochondrial protein abundance were abolished following the exercise intervention as exercise increased the abundance of various mitochondrial proteins, including eight NADH dehydrogenase subunits (encoded by *NDUFB3, NDUFC2, NDUFS6, NDUFA6, NDUFA7, NDUFA2, NDUFA8*, and *NDUFA5*) [137].

Likewise, as mentioned above, Robinson et al. took advantage of a DDA label-free proteomic approach and LC-MS/MS to identify age-related changes in the proteome as well as the effect of exercise in older individuals (age 65–80 years) when compared to a younger group (age 18–30 years) of study participants [143]. Endurance exercise training is known to improve both insulin sensitivity and the mitochondrial oxidative capacity [93,144]. Consistently, the proteomic analysis revealed that 12 weeks of high-intensity aerobic interval training (HIIT) exercise increased the number of mitochondrial proteins quantified in the skeletal muscle of both the younger and the older group, with the increase being the largest in the older study participants’ (25 and 169) proteins [143]. In the older group, HIIT particularly upregulated proteins in pathways that are reflective of a mitochondrial oxidative phenotype, thus underlining the improved aerobic capacity identified following the HIIT exercise intervention [143].

In line with these findings, Ubaida-Mohien et al. used LC-MS/MS to quantify the human skeletal muscle proteome from 60 well-characterized healthy individuals aged 20–87 years, who self-reported their level of physical activity [153]. Age-related changes in the skeletal muscle proteome of the same study population were described in a separate publication and are presented in a previous section. In the present study, TMT labeling was used for quantification, after which the whole-tissue muscle samples were analyzed using a nano-LC-MS/MS setup [153]. Approximately 4300 proteins were identified, of which 1019 proteins were significantly associated with physical activity [153]. Mitochondrial proteins constituted roughly 15% of all detected proteins and the abundance of about 40% of the mitochondrial proteins was significantly associated with the level of self-reported physical activity [153]. Approximately 75% of the mitochondrial proteins that were differentially regulated by self-reported physical activity were positively associated with higher physical activity. Among the mitochondrial proteins showing a positive association between protein abundance and self-reported physical activity, there were numerous OXPHOS subunits, 14 TCA cycle-related proteins, and 10 mitochondrial solute carriers [153]. The higher abundance of mitochondrial proteins in aging individuals with a high level of self-reported physical activity was independent of age, thus supporting the findings in all other studies presented in this section that physical exercise may prevent, or at least postpone, the age-related decline in mitochondrial function. These changes seem to occur independent of the exercise intervention investigated, and given that the number of regulated mitochondrial proteins are reflective of the mitochondrial function, the consistency of these findings strengthens the conclusion that exercise is of great importance in order to maintain a healthy mitochondrial population during aging. Whether the same improvements in the mitochondrial proteome occur in the muscle of obese and T2D individuals has only been investigated in a single study [103], which only identified a relatively low number of proteins when considering what we would expect today given the recent advances in MS-based proteomics.

## 5. Concluding Remarks and Perspective

The currently available data from quantitative MS-based proteomic studies of human skeletal muscle clearly support the notion that mitochondrial dysfunction is linked to IR in obesity, T2D, and aging. However, the data presented also suggest that at least the aging-associated alterations in the mitochondrial proteome may be prevented, or at least postponed, by regular aerobic exercise. Whether exercise training has the same beneficial effect on the muscle mitochondrial proteome in patients with T2D as in healthy individuals remains to be further explored. The characterization of exercise-mediated changes in the phosphoproteome revealed that acute exercise mainly mediates its effects through altered PTMs and a future study aiming to characterize how exercise influences the mitochondrial phosphoproteome or acetylome would therefore also be of interest and could provide a further understanding as to whether and how exercise may help prevent, or at least postpone, the development of IR and T2D.

The findings presented in the present review also clearly show that the use of subfractionated samples, either using isolated single muscle fibers or isolated mitochondria, has the potential to markedly increase the information gained from a proteomic study. In particular, the use of isolated mitochondria enables a deeper characterization of the mitochondrial proteome and any PTMs of interest and could therefore contribute to an increased understanding of how mitochondria respond to both short-term and chronic metabolic changes.

Combined, given the recent advances in MS-based proteomics as well as in the bioinformatics tools available for data analysis, we expect that future proteomics studies of human skeletal muscle and isolated mitochondria will lead to an immense increase in the number of proteins and the PTMs identified and quantified, thus obtaining a much larger coverage of the mitochondrial proteome. This will provide the basis for further mechanistic studies into the mitochondrial function in healthy skeletal muscle but also the role of mitochondrial dysfunction in insulin-resistant conditions, such as obesity, T2D, and aging.

## Abbreviations

1-DE	One-dimensional gel electrophoresis
2D-DIGE	Two-dimensional difference gel electrophoresis
2-DE	Two-dimensional gel electrophoresis
DDA	Data dependent analysis
DIA	Data independent analysis
ESI	Electrospray ionization
HIIT	High intensity interval training
HILIC	Hydrophilic interaction chromatography
HPLC	High performance liquid chromatography
IMAC	Immobilized metal affinity chromatography
IMF	Intermyofibrillar
IR	Insulin resistance
MALDI	Matrix-associated laser desorption ionization
MS	Mass spectrometry
MS/MS	Tandem mass spectrometry
NSAF	Normalized spectral abundance factor
OXPHOS	Oxidative phosphorylation
PGC	Proliferator-activated receptor γ co-activator
PRM	Paralleled reaction monitoring
PTM	Post-translational modification
SRM	Selected reaction monitoring
SS	Subsarcolemmal
SWATH	Sequential window acquisition of all theoretical mass spectra
T2D	Type 2 diabetes
TCA	Tricarboxylic acid
TMT	Tandem mass tag
TOF	Time-of-flight
